# Prevalence of self-reported comorbidities in HIV positive and HIV negative men who have sex with men over 55 years—The Australian Positive & Peers Longevity Evaluation Study (APPLES)

**DOI:** 10.1371/journal.pone.0184583

**Published:** 2017-09-08

**Authors:** Kathy Petoumenos, Robin Huang, Jennifer Hoy, Mark Bloch, David J. Templeton, David Baker, Michelle Giles, Matthew G. Law, David A. Cooper

**Affiliations:** 1 Kirby Institute, UNSW Sydney, Sydney, New South Wales, Australia; 2 Department of Infectious Diseases, The Alfred Hospital and Monash University, Melbourne, Victoria, Australia; 3 Holdsworth House Medical Practice, Darlinghurst, New South Wales, Australia; 4 RPA Sexual Health, Sydney Local Health District and Central Clinical School, The University of Sydney, Camperdown, New South Wales, Australia; 5 East Sydney Doctors, Darlinghurst, New South Wales, Australia; 6 Department of Infectious Diseases, Monash Health, Melbourne, Victoria, Australia; San Antonio Military Medical Center, UNITED STATES

## Abstract

In Australia, almost half of HIV-positive people are now aged over 50 and are predominately gay and bisexual men (GBM). Compared to the general HIV-negative population, GBM engage more in behaviours that may increase the risk of age-related comorbidities, including smoking, high alcohol consumption and recreational drug use. The objective of APPLES was to compare comorbidities and risk factors in HIV-positive older GBM with an appropriate control group of HIV-negative GBM. We undertook a prospectively recruited cross-sectional sample of HIV-positive and HIV-negative GBM ≥ 55 years. Detailed data collection included clinic data, a health and lifestyle survey, and blood sample collection. We report key demographic, laboratory markers and self-reported comorbidities by HIV status. For selected comorbidities we also adjust HIV status *a priori* for age, smoking and body mass index. Over 16 months 228 HIV-positive and 218 HIV-negative men were recruited. Median age was 63 years (IQR: 59–67). Although more HIV-positive men reported having ever smoked, smoking status was not statistically different between HIV positive and HIV negative men (p = 0.081). Greater alcohol use was reported by HIV-negative men (p = 0.002), and recreational drug use reported more often by HIV-positive men (p<0.001). After adjustment, HIV-positive men had significantly increased odds of diabetes (adjusted Odds ratio (aOR): 1.97, p = 0.038), thrombosis (aOR: 3.08, p = 0.007), neuropathy (aOR: 34.6, P<0.001), and non-significantly increased odds for heart-disease (aOR: 1.71, p = 0.077). In conclusion, HIV-positive GBM have significantly increased odds for key self-reported comorbidities. This study underscores the importance of an appropriate HIV-negative control group for more accurate evaluation of the risk and attribution of age-related comorbidities in HIV-positive people.

## Introduction

More than 50% of the estimated 25,000 people living with HIV (PLHIV) in Australia are estimated to be over 50 years of age [1]. Similar trends are being observed in the US and Europe [2]. New challenges have emerged among this ageing population. In developed countries, most HIV positive people on antiretroviral therapy (ART) now achieve durable suppression of HIV replication. Immunodeficiency is now uncommon and AIDS defining conditions rare. Overall, PLHIV have vastly improved long-term clinical outcomes and overall survival [3–10]. Nonetheless, despite life-expectancy approaching that of the general population [11–14], survival rates among PLHIV remain poorer than that of the general population, with larger seen disparities among specific subpopulations including smokers, alcohol and drug users, and individuals of lower socio-economic status [12, 15].

Despite the clear success of ART, PLHIV appear to have an increased risk of serious non-communicable diseases (NCDs) (also referred to as serious non-AIDS events) including cardiovascular, liver and kidney disease, malignancies and bone disorders [16, 17]. In fact, an increased risk of a number of age-related NCDs in PLHIV compared to HIV negative populations has been reported across several studies internationally. Earlier studies suggested an increased risk of age-related comorbidities occurring earlier (at a younger age) in PLHIV [18, 19]. More recent studies suggest an increase in NCDs overall, but with little or no age association [20, 21]. However, causes of these age-related NDCs are an intricate mix of HIV disease itself, ART, genetic factors, co-infections such as hepatitis C (HCV) and hepatitis B virus (HBV), lifestyle factors including cigarette smoking and alcohol use [22, 23], as well as persistent chronic inflammation/ immune activation despite suppressed HIV replication [16, 24–28]. Disentangling of the impact of ART, and HIV infection from lifestyle factors is important in understanding the true risk of these NCDs among HIV positive people. The few cohort studies with HIV positive and well matched HIV negative controls report a greater prevalence of NCDs among older (>45 years) HIV positive people compared with HIV negative people [29] [30].

We established the Australian Positive & Peers Longevity Evaluation Study (APPLES) to investigate the prevalence of age-related comorbidities and risk factors among older HIV positive and HIV negative men. In this paper we report a cross-sectional comparison of the prevalence of self-reported comorbidities and health and behavioural risk factors.

## Materials and methods

APPLES was a prospectively recruited cross-sectional sample of HIV positive and HIV negative men who identify as gay or bisexual men (GBM), aged 55 years and over. Recruitment to APPLES commenced in August 2014, with a recruitment target of 450 (225 HIV positive and 225 HIV negative) GBM. HIV negative men were required to have had a negative HIV antibody test within the last 12 months. There was no restriction in terms of when HIV negative men last reported having had sex with other men.

### Recruitment

Participants were recruited from a subset of sites from the existing Australian HIV Observational Database (AHOD) network [31], and included general practice (GP), sexual health clinics (SHC), and tertiary referral hospitals from most states and territories in Australia. Potentially eligible participants were identified by the site principal investigator (PI) or study co-ordinator and invited to participate. The study was also advertised by brochures placed at clinic reception, and further advertisements via the AIDS Council of New South Wales (ACON) and Facebook.

Following informed consent, sites recorded participant details (age and HIV status) onto a secure APPLES specific electronic case record form (eCRF) that notified the Kirby Institute investigators of a newly recruited participant. Participant full name (required) and address (optional) were also recorded, though these data were stored separately to ensure participant confidentiality. These identifying details were requested from participants for future data linkage to state and national health registries.

### Data collection

Lifestyle and behaviour survey: Participants were asked to self-complete a questionnaire similar to the *45 and Up Study* questionnaire for men [32] addressing health and lifestyle factors such as personal and family medical history, smoking (including age start and stop) and alcohol use, exercise and diet. Additional questions on recreation drug use were included. [Note: Smoking pack-years ((average cigarettes per day/20)*number of years smoked) and smoking burden (calculation: average pack-years/total population with smoking status reported (non-smokers contributing 0 pack-years)) were subsequently calculated]. The questionnaire also included questions regarding diagnosis of specific comorbidities (including heart disease, diabetes, thrombosis, high blood pressure, cancer, among others) at any time and age at diagnosis. Participants were asked to record recent (within the last four weeks) treatment for specific conditions (including cancer, “blood clot”, depression, high blood cholesterol, osteoarthritis or osteoporosis, among others). The questionnaire was online, however, for participants who did not have access to a computer or the internet, a paper copy was made available. For participants indicating they would complete the survey online, a reminder email was sent if the questionnaire had not been completed within five days of their study visit.

Clinical data: Recruiting sites recorded the following data on the eCRF—anthropometric measures, medications to manage comorbidities (lipid lowering, diabetes, cardiovascular disease, hypertension, and depression), laboratory measures (lipids (Total cholesterol (TC), low density lipoprotein cholesterol (LDL), high density lipoprotein cholesterol (HDL), triglycerides (TG)), glucose, haemoglobin, creatinine, alanine aminotransferase (ALT), aspartate aminotransferase (AST), Prostate Specific Antigen (PSA), vitamin D), and hepatitis B and hepatitis C infection status. These data were obtained from the patient medical records and the most recent measure was recorded. For HIV positive participants, additional data recorded included current ART, most recent CD4+ count and HIV viral load, and whether participants have ever been diagnosed with an AIDS defining illness. Blood samples for assessment of specific biomarkers (including D-dimer, hsCRP, cystatin C, and IL-6) and storage for future research testing were also collected from all participants.

### Ethical considerations

This study was approved by St Vincent’s Hospital, Sydney Human Research Ethics Committee, and all other institutional review boards as required by participating sites. Participants were required to provide written informed consent to take part in the study. Consent was specifically sought to record clinic data, complete the online questionnaire, record full name for data linkage, collection and storage of blood samples, and to be contacted in the future regarding participation in related research studies.

### Statistical analysis

Data from the study eCRF and the self-completed questionnaire were summarised by HIV status. Descriptive statistics were used to summarise participant demographics, anthropometry, risk and comorbidity by HIV status. Study groups were compared using the χ2, Wilcoxon rank-sum, nonparametric test for trend, or Student t test as appropriate. Logistic regression methods were used to assess self-reported comorbidity by HIV status adjusted *a priori* for age, smoking status and BMI. All reported P values are 2-sided and a value less than 0.05 was considered statistically significant. For these outcomes we also undertook a sensitivity analysis limiting the population to participants recruited via general practice only, for whom the reason for attendance at the GP clinics might be for reasons other than sexually transmitted infection (STI) or HIV testing or management.

## Results

### Recruitment

A total of 446 (228 HIV positive and 218 HIV negative) GBM aged 55 years and over were recruited to APPLES. The recruitment target for HIV positive men was achieved within nine months. The rate of recruitment for HIV negative men was slower, taking more than 16 months, with the decision to cease recruitment when 218 HIV negative men had been recruited. Among the recruited participants 200 (88%) HIV positive men and 189 (87%) HIV negative men completed the online survey.

### Demographic and lifestyle characteristics

Table 1 includes basic demographic data for the cohort. The majority (62%) of participants were recruited via general practices (GP), followed by sexual health clinics (21%). Most HIV negative men were recruited via GP (70%) and sexual health clinics (24%), whilst HIV positive men were largely recruited via GP (48%) or tertiary hospitals (33%). HIV positive men were slightly younger than HIV negative men (median (IQR): 62 years (58–66) vs 65 years (60–69)). The majority of HIV positive (69%) and HIV negative (68%) men were born in Australia, and 40% of HIV positive and 41% of HIV negative men were employed.

**Table 1:** Participant demographic and anthropometric measures by HIV status.

	HIV negative (n = 218)	HIV positive (n = 228)
**Recruitment Sites**		
General practitioner (GP)	156 (71.6%)	110 (48.2%)
Sexual health clinic (SH)	52 (23.8%)	44 19.3%)
Tertiary hospital (TR)	10 (4.6%)	74 (32.5%)
**Age (years)**		
Median (IQR)	65 (60–69)	62 (58–66)
≤60	69 (31.7%)	94 (41.2%)
61–70	114 (52.3%)	110 (48.3%)
71–80	31 (14.2%)	23 (10.1%)
>80	4 (1.8%)	1 (0.4%)
**Height (cm)**		
Median (IQR)	176 (172–181)	176 (172–180)
**BMI (kg/m**^**2**^**)**		
Underweight (<18.5)	0 (0%)	0 (0%)
Healthy weight (18.5 to 24.9)	69 (33.2%)	92 (42%)
Overweight (25 to 29.9)	89 (42.8%)	96 (43.8%)
Obese (>30)	50 (24%)	31 (14.2%)
**Waist circumference (cm)**		
< 80	8 (4.4%)	6 (3.5%)
80–100	88 (49%)	91 (53.9%)
101–120	76 (42.2%)	66 (39.1%)
> 120	8 (4.4%)	6 (3.5%)
**HBV surface antigen positive**		
Yes	3 (2.7%)	7 (5%)
No	110 (97.3%)	133 (95%)
**HCV antibody positive**		
Yes	1 (0.9%)	13 (8%)
No	103 (99.1%)	150 (92%)
***Data from self-completed questionnaire***	[n = 189]*	[n = 200]*
**Country of Birth**		
Australia	130 (68.8)	138 (69)
New Zealand	12 (6.3)	11 (5.5)
United Kingdom	20 (10.6)	19 (9.5)
Other	27 (14.3)	32 (16)
**Education**		
University degree or higher	106 (56.1)	86 (43.4)
Certificate/diploma	28 (14.8)	28 (14.1)
Trade/apprenticeship	7 (3.7)	19 (9.6)
Higher school or leaving certificate	30 (15.9)	40 (20.2)
School or intermediate certificate**	11 (5.8)	16 (8.1)
No school certificate	7 (3.7)	9 (4.5)
**Employment**		
Employed	78 (41.3)	80 (40.0)
Retired	96 (50.8)	90 (45.0)
Unemployed	15 (7.9)	30 (15.0)
**Insurance**		
Private health insurance (with extras)	109 (58.9)	71 (36)
Private health insurance (without extras) ***	13 (7.0)	9 (4.6)
Health care concession card	41 (22.2)	74 (37.6)
Department of Veterans’ Affairs****	1 (0.5)	2 (1.0)
None of the above	21 (11.4)	41 (20.8)

*Number completed questionnaire;

** qualification issued by the Board of Studies typically at the end of four years of high school studies;

*** Private insurance with extras—hospital cover with additional options such as dental treatment, home nursing, podiatry, physiotherapy etc.

****health cards to eligible veterans and former members of Australia's defence force, their widows/widowers and dependants

Whilst smoking status overall was not significantly different by HIV status (p = 0.081), more HIV positive men had a history of ever smoking (prior or current smokers) compared to HIV negative men (60% vs 49%). Similarly, HIV positive men had a greater overall burden of smoking as measured by life-time pack years (median 6 years (IQR: 0–30.0) compared to a median of 0 years (IQR: 0–22.5) among HIV negative men, although this difference also did not reach statistical significance (p = 0.072). Greater alcohol use was reported by HIV negative men (p = 0.002), and recreational drug use reported more often by HIV positive men (42% vs 24%; p<0.001). Proportionally more HIV positive men (4%) also report ever having injected drugs compared to HIV negative men (2%), although not statistically significant. HIV positive and HIV negative men reported similar hours of exercise per week (Table 2).

**Table 2:** Participant self-report lifestyle behaviours by HIV status*.

	HIV negative (n = 189)	HIV positive (n = 200)	p-value
**Smoking status**	**N (%)**	**N (%)**	
Current	20 (11.3)	21 (11.2)	0.081
Prior	66 (37.3)	91 (48.4)	
Never	91 (51.4)	76 (40.4)	
	**Median (IQR)**	**Median (IQR)**	
Smoking pack-years (ever smokers)**	24.1 (10.6–42.9)	27.6 (10.0–41.3)	
Overall smoking burden***	0 (0–22.5)	6.5 (0–30.0)	0.072
**Number of Alcohol drinks per week**	6 (1–14)	3 (0–10)	0.002
**Number of drinking days per week**	**N (%)**	**N (%)**	
0	40 (22.2)	61 (32.3)	0.042
1	11 (6.1)	15 (7.9)	
2	7 (3.9)	12 (6.4)	
3+	122 (67.8)	101 (53.4)	
**Recreational drug use**			
No	142 (75.9)	115 (58.1)	<0.001
Yes	45 (24.1)	83 (41.9)	
**Injecting drug use**			
No	183 (98.9)	188 (95.9)	0.106
Yes	2 (1.1)	8 (4.1)	
**Exercise**	**Median (IQR)**	**Median (IQR)**	
Hours walked per week	3.4 (2–7)	3 (1.5–6.3)	0.511
Hours exercised moderately per week	3 (1–6)	3 (1–6)	0.753
Hours exercised vigorously per week	0.5 (0–2)	0 (0–2.5)	0.923

*data from self-completed questionnaire;

**Pack-years calculation: (average cigarettes per day/20)*number of years smoked;

*** Overall smoking burden calculation: average pack-years/total population with smoking status reported (non-smokers contribute 0 pack-years)

### Clinical characteristics and self-reported comorbidities

More HIV positive men were of healthy body mass index (BMI) (42%) compared with HIV negative men (33%), and a greater proportion of HIV negative men were obese (HIV negative 24% compared with 14% of HIV positive men). Hypertension (defined as systolic >140/diastolic >90 or on antihypertensive medication) was similar for HIV positive and HIV negative men (61% vs 57%, p = 0.445). A large proportion of both HIV positive (74%) and HIV negative (61%) men were receiving concomitant medications (including anti-platelet agents (17% vs 19%, p = 0.554), ace-inhibitors (15% vs 18%, p = 0.642), lipid lowering drugs (44% vs 29%, p = 0.001), antidiabetic agents (13% vs 7%,p = 0.059, anabolic steroids (5% vs 2%, p = 0.037) or antidepressants (22% vs 15%, p = 0.065) respectively).

A significantly greater proportion of HIV negative men had higher LDL-cholesterol levels >3.5 mmol/L (30% vs 18%, p = 0.008) and HDL-cholesterol levels ≥1.0 mmol/L (88% vs 77%, p = 0.005) compared to HIV positive men, whilst a greater proportion of HIV positive men had elevated triglycerides > 2 mmol/L compared to HIV negative men (41% vs 19%, p<0.001). There were no significant differences between HIV positive and HIV negative in regards to total cholesterol (TC) or TC: HDL ratio. ALT and AST levels were significantly different for HIV positive compared to HIV negative men, with 25% of HIV positive compared to 14% of HIV negative men with an ALT ≥40 U/L (p = 0.004), and 12% of HIV positive compared to 5% of HIV negative men with an AST ≥40 U/L (p = 0.014).

In this sample, evidence of past or current hepatitis C virus (HCV) coinfection was significantly greater among HIV positive men (8%) compared to HIV negative men (1%), whilst only marginally greater proportion of HIV positive men were reported to be hepatitis B virus (HBV) surface antigen positive compared with HIV negative men (5% vs 3%) (Table 1).

The majority of the HIV positive men (44%) were diagnosed with HIV prior to 1991, 25% were diagnosed between 1991 and 2000, and a further 25% between 2001 and 2010. Twelve participants (6%) were diagnosed with HIV since 2011. All but three HIV positive participants were currently on ART at the time of recruitment, median CD4+ count (IQR) was 620 (453–812), and 98% had an HIV viral load less than 200 copies/mL at last measurement (Table 3).

**Table 3:** Characteristics of HIV positive participants.

	HIV positive (n = 228)
**Year of HIV diagnosis**	
1981–1990	87 (43.7%)
1991–2000	50 (25.1%)
2001–2010	50 (25.1%)
2011–present	12 (6.1%)
**Median years (IQR) since diagnosis**	22.3 (12.3–22.9)
**CD4 T lymphocyte count (cells/μL)**	
Median (IQR)	619 (453–812)
< 200	8 (3.5%)
200–349	21 (9.3%)
350–500	39 (17.2%)
> 500	159 (70%)
**HIV plasma viral Load**	
Detectable	6 (2.6%)
Undetectable (<200 copies/mL)	222 (97.4%)
**Antiretroviral therapy (ART) history**	
ART naive	2 (0.9%)
Currently on ART	225 (98.7%)
Past but now stopped	1 (0.4%)

**Fig 1:**
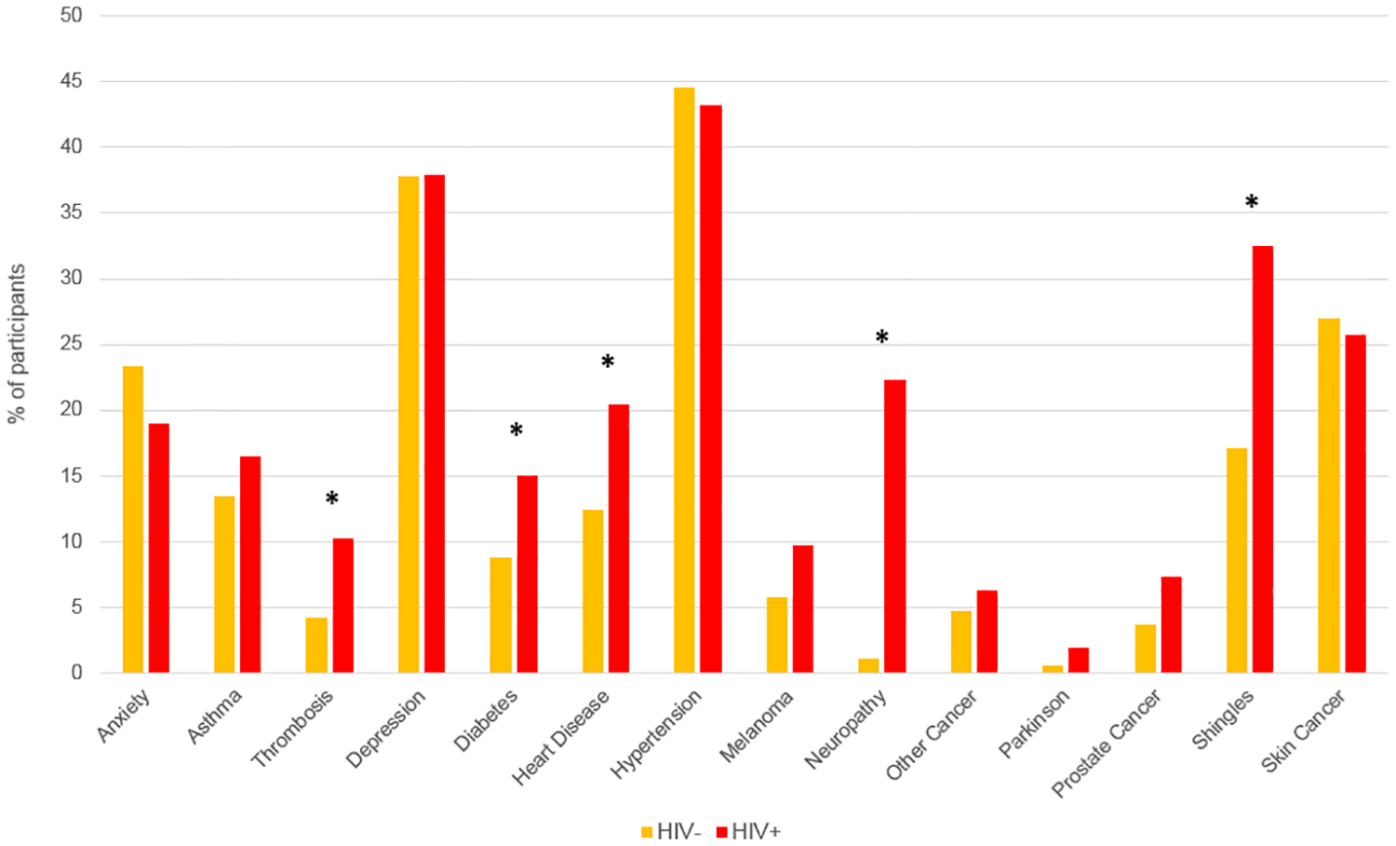
Selected self-reported comorbidities by HIV status. *denotes (unadjusted) P-value < 0.05 **hypertension—self reported “high blood pressure”.

Table 4 and Fig 1 show selected self-reported comorbidities by HIV status. HIV positive men reported higher rates of thrombosis (10.5% vs 4.2%; p<0.019), diabetes (15% vs 9%; p = 0.069), heart disease (20% vs 12%; p = 0.048), neuropathy (23% vs 1%; p<0.001), and shingles (32.5% vs 16.9%; p<0.001). After adjustment for age, smoking status and BMI, HIV positive men had significantly increased odds of diabetes (Adjusted Odds ratio (aOR (95% confidence interval (CI))): 1.97 (1.04–3.75), p = 0.038), thrombosis (aOR (95%CI): 3.08 (1.36–6.98), p = 0.007), neuropathy (aOR (95%CI): 34.6 (8.88–134.47), P<0.001), shingles (aOR (95%CI): 2.32 (1.41–3.83), p = 0.001) and non-significantly increased odds for heart disease (aOR (95%CI 0.94–3.1): 1.71, p = 0.077). For the remaining comorbidities there was no significant difference in crude rates between HIV positive and HIV negative men, however, once adjusted for smoking, age and BMI, a significantly elevated risk for HIV positive men for melanoma (aOR (95%CI): 2.20 (1.03–4.68), p = 0.041) and prostate cancer (aOR (95%CI): 2.78 (1.11–6.95), p = 0.028) was observed. A significantly greater proportion of HIV positive men (11%) also reported taking medication in the last 4 weeks for osteoporosis and bone disease compared to HIV negative men (3.2%), with a 3.8-fold increased odds after adjustment for age, smoking status and BMI (p = 0.005) (data not shown).

**Table 4:** Unadjusted and adjusted odds for selected self-reported comorbidities (HIV positive compared to HIV negative men).

	HIV-	HIV+	Unadjusted	p-value	Adjusted**	p-value
	%	%	OR (95%CI)*		aOR (95%CI)*	
**Anxiety**	23.0	19.0	0.75 (0.46–1.22)	0.247	0.67 (0.4–1.11)	0.119
**Asthma**	13.8	15.5	1.15 (0.65–2.02)	0.627	1.09 (0.61–1.95)	0.780
**Thrombosis**	4.2	10.5	2.56 (1.12–5.82)	0.025	3.08 (1.36–6.98)	0.007
**Depression**	38.1	37.5	0.98 (0.65–1.47)	0.904	0.84 (0.54–1.3)	0.430
**Diabetes**	9.0	15.0	1.79 (0.95–3.36)	0.072	1.97 (1.04–3.75)	0.038
**Heart Disease**	12.2	19.5	1.75 (1–3.06)	0.050	1.71 (0.94–3.1)	0.077
**Hypertension**	45.0	43.5	0.94 (0.63–1.41)	0.770	1.17 (0.75–1.81)	0.491
**Melanoma**	5.8	10.0	1.8 (0.84–3.86)	0.133	2.2 (1.03–4.68)	0.041
**Neuropathy**	1.1	22.5	21.95 (6.02–80.1)	<0.001	34.6 (8.9–134.5)	<0.001
**Other Cancer**	4.8	6.0	1.26 (0.53–3.01)	0.603	1.3 (0.55–3.07)	0.556
**Parkinson's disease**	0.5	2.0	2.88 (0.45–18.55)	0.266	2.47 (0.61–10.09)	0.207
**Prostate Cancer**	3.7	7.0	1.89 (0.76–4.69)	0.168	2.78 (1.11–6.95)	0.028
**Shingles**	16.9	32.5	2.34 (1.45–3.79)	0.001	2.32 (1.41–3.83)	0.001
**Skin Cancer**	25.9	25.5	0.98 (0.62–1.54)	0.922	1.18 (0.73–1.93)	0.497
**Syphilis**	17.5	36.5	2.69 (1.68–4.32)	<0.001	2.86 (1.73–4.71)	<0.001

*Reference group—HIV negative men

** Adjusted for age, smoking status, and BMI

**Fig 2:**
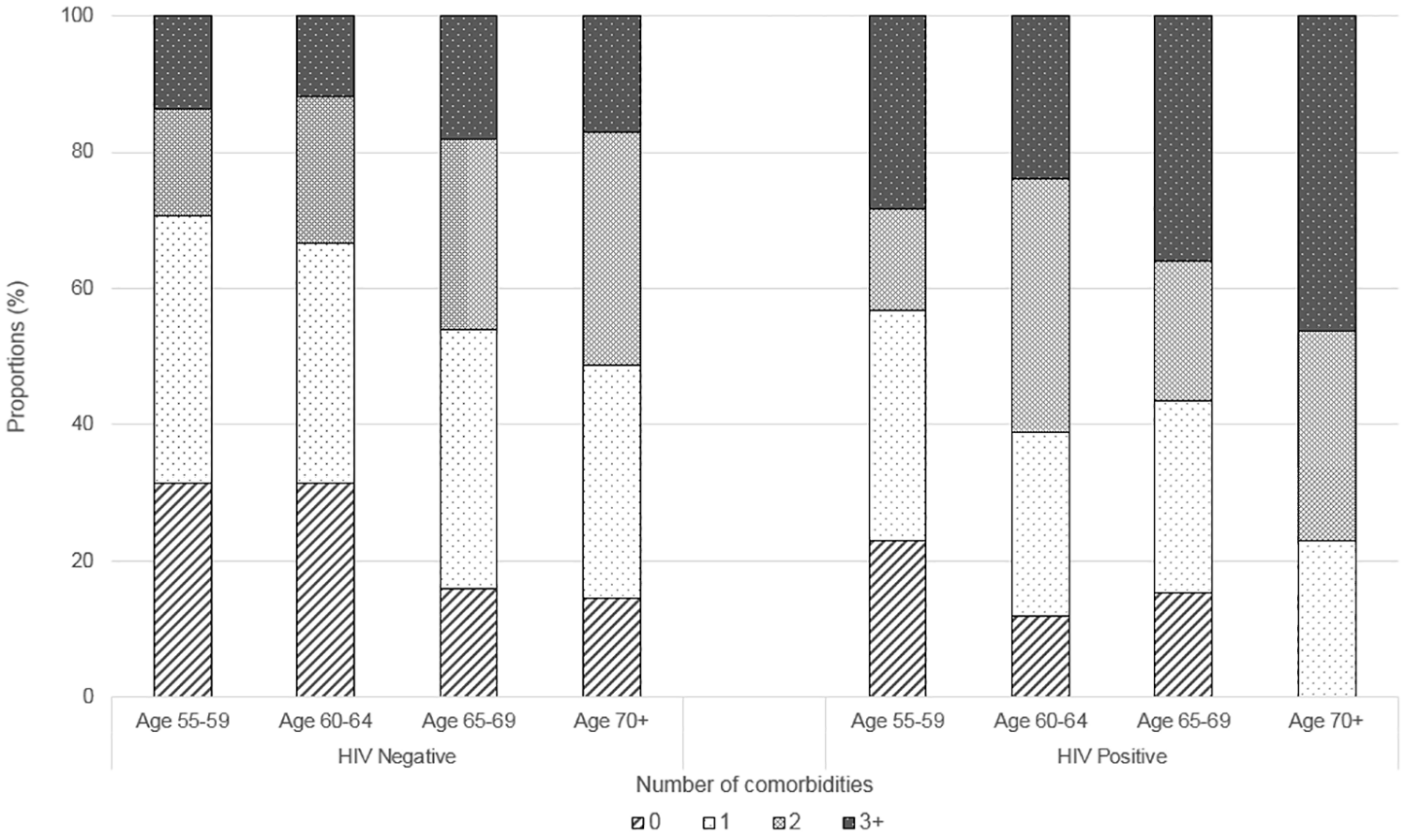
Number of comorbidities by HIV status and age group.

Fig 2 illustrates the proportion of HIV positive and HIV negative men with 0, 1, 2 or 3+ comorbidities, stratified by age group. For both groups, multi-morbidity became more prevalent with increasing age. HIV positive men reported a significantly higher mean number of comorbidities compared to HIV negative men (p = 0.002). Eighty-five percent of HIV positive men reported one or more comorbidities, and just over half (56%) reported two or more comorbidities. Of the HIV negative men, 77% reported one or more co-morbidities, with 39% reporting two or more comorbidities.

### Sensitivity analysis

Among the participants recruited through GP sites only, HIV positive men reported similar high rates of previous smoking, recreational drug use and lower rates of alcohol use as HIV negative men. Higher rates of thrombosis (9% vs 5%) and heart disease (17% vs 14%) were still observed among HIV positive men (though no longer statistically significant), and similar rates of diabetes (HIV positive 12% vs HIV negative 9%, p = 0.563) were recorded. As with the full cohort, significantly more (p = 0.043) HIV negative men reported LDL-cholesterol levels above 3 mmol/L (31% vs 18%) and HDL-cholesterol levels ≥ 1 mmol/L (89% and 76%) compared to HIV positive men.

## Discussion

In this sample of HIV positive and HIV negative GBM over the age of 55 years, an increased prevalence of several self-reported comorbidities including thrombosis, diabetes, heart disease, neuropathy and bone disease was reported by HIV positive men compared to HIV negative men. HIV positive men also reported a significantly increased mean number of comorbidities compared to HIV negative men. Lifestyle risk factors such as ever having smoked and use of recreational drugs were more common among HIV positive men, while HIV negative men more often reported alcohol use. These data suggest that older HIV positive men experience some NCDs at higher rates compared to HIV negative GBM of similar age, despite similar rates of smoking and other traditional risk factors.

Our findings are consistent with the AGEhIV cohort [29], one of very few other studies with appropriate age-matched HIV negative controls established prospectively to address questions around ageing. In a cross-sectional comparison for a number of age related NCDs in the AGEhIV cohort, HIV positive people experienced a significantly greater number of comorbidities compared to HIV negative controls, as well as a significant increased prevalence of myocardial infarction, peripheral arterial disease, impaired renal function [29], and osteoporosis among HIV positive compared to HIV negative individuals [33]. Increased prevalence of traditional risk factors among HIV positive populations (such as smoking, elevated lipids, hyperglycaemia, altered body composition, alcohol and recreational drug use) significantly contribute to the increased risk for many of these NCDs [34–36]. However, even after adjustment for some of these traditional risk factors, we found the odds of reporting comorbidities including thrombosis, diabetes and to a lesser extent heart disease to be elevated among HIV positive men. Smoking is one of the most important modifiable traditional risk factors for NCDs and accounts for considerable excess mortality and morbidity (1.5–2 fold or greater), in particular for CVD and non-AIDS cancers among HIV positive people [37–41]. Smoking rates across HIV populations range 40 to 70% [42–45] and are often higher among sub-populations, including GBM. In our study almost 50% or more of both HIV positive and HIV negative men reported ever smoking.

Dyslipidemia is particularly common among HIV positive people on ART with well documented associations with both protease inhibitor (PI) and nucleoside reverse transcriptase inhibitor (NRTI) use [46]. In our sample of older HIV positive people with an median duration of HIV disease of 20 or more years, the majority would have at some time been exposed to earlier generation NRTI- and PI-based ART that is associated with greater dyslipidaemia than contemporary ART, and had longer duration of exposure to such ART. In our study, we observed higher LDL-cholesterol levels among HIV negative men which may be a result of more frequent testing and resultant management of lipid abnormalities among HIV positive people. Although more than three quarters of HIV positive men had high HDL levels reported, significantly more HIV positive men had lower HDL cholesterol compared to HIV negative men. HIV infection is associated with low HDL-cholesterol[47], and low HDL has been identified as an independent risk factor for cardiovascular disease [48]. Improved management of dyslipidaemia to reduce the elevated risk of cardiovascular disease related to low HDL levels among PLHIV could further improve their cardiovascular risk profile.

ART use has been associated with several other age-related comorbidities, including hyperglycaemia, CVD, thrombosis, diabetes and bone disease. Cardiovascular and related diseases may be driven in part by increases in lipids, with PI use having the most profound effect on triglyceride, TC and LDL cholesterol levels [46]. PI and tenofovir disoproxil fumarate use [49–51], have been associated with reduced bone mineral density, and tenofovir use has also been linked to renal disease [51]. It is likely that increased screening for such comorbidities in HIV positive men results in more of these comorbidities being identified and appropriately managed.

There are limitations to our study. First, the cross-sectional nature of this study can only demonstrate an association rather than causation. Comorbidities were self-reported and therefore subject to recall bias, and for some diseases, notably heart disease, there was no strict definition of what form of heart diseases this included. Further, it was not feasible to cross-check with clinic records as many of these events may have occurred many years ago in some cases, and not necessarily available at the clinics the patients are currently seen. However, as these data were reported and collected by similar means for both HIV positive and HIV negative men, this recall bias should not be differential. We also based our questions on an established questionnaire to investigate ageing, used in a large state wide population based cohort study of more than 200,000 people over the age of 45 years [32], thereby allowing our data to be compared with a population based cohort. Second also due to the cross-sectional nature of our study, we were unable to establish if any of these diseases were increasing or decreasing over time. Such trends could be an outcome of temporal changes in availability of better tolerated, more lipid-friendly ART and also updated HIV management guidelines, such as more routine screening for, and aggressive management of, modifiable risk factors for NCDs. Third, HIV negative participants were not population based controls, but rather recruited via the same treatment clinics from where the HIV positive men were enrolled, and therefore the potential for sampling bias. As the majority were recruited from GPs, it is possible that the negative men in our study are more unwell and not representative of all HIV negative GBM. However, Australian sexually transmitted infection (STI) guidelines [52]recommend that GBM have STI and HIV testing at least annually, and many of the HIV negative men recruited to our study may have been attending the clinic for testing rather than for any other underlying clinical condition. Further, as part of this study we also collected blood samples for storage which would be more challenging with a population based sample. Finally, we also limited our study to GBM, so our findings would not be generalisable to PLHIV from other subgroups of the population.

In conclusion, several age-related comorbidities are more prevalent among older HIV positive men, even after adjustment for traditional risk factors. Despite effective ART, morbidity among HIV positive individuals will continue to put pressure on health services due to increasing NCD in an expanding and ageing HIV positive population. Changes in the morbidity profile of ageing HIV positive people has considerable implications for treatment, as well as policy and planning of health services for these people. Understanding the relative contribution of HIV infection, ART and lifestyle factors to the development of these comorbidities in HIV positive people is imperative to develop screening, prevention and advocacy programs for the rapidly expanding older population of Australian PLHIV.
